# Seasonal Malaria Chemoprevention Therapy in Children Up To 9 Years of Age: Protocol for a Cluster-Randomized Trial Study

**DOI:** 10.2196/51660

**Published:** 2024-01-22

**Authors:** Mahamoudou Toure, Jeffrey G Shaffer, Daouda Sanogo, Soumba Keita, Moussa Keita, Fousseyni Kane, Bourama Traore, Djeneba Dabitao, Aissata Kone, Cheick Oumar Doumbia, Joseph Keating, Joshua Yukich, Helle H Hansson, Alyssa E Barry, Mahamadou Diakité, Michael Alifrangis, Seydou Doumbia

**Affiliations:** 1 University Clinical Research Center Universite des Sciences, des Techniques et des Technologies Bamako Mali; 2 Tulane University School of Public Health and Tropical Medicine New Orleans, LA United States; 3 Mali National Malaria Control Program Bamako Mali; 4 Center for Medical Parasitology, Department of Immunology and Microbiology University of Copenhagen Copenhagen Denmark; 5 Institute for Mental and Physical Health and Clinical Translation (IMPACT) and School of Medicine, Deakin University, Geelong Melbourne Australia; 6 Life Sciences Discipline, Burnet Institute Melbourne Australia

**Keywords:** malaria, seasonal malaria chemoprevention, RCT, randomized, controlled trial, controlled trials, parasite, parasites, mosquito, mosquitoes, vector-borne, malarial, antimalarial, age, Plasmodium falciparum, protocol, cluster-randomized trial, child, children, infant, infants, pediatric, pediatrics, clinical trial, clinical trials, drug, drugs, pharmacy, pharmacology, pharmaceutic, pharmaceutics, pharmaceuticals, pharmaceutical, medication, medications

## Abstract

**Background:**

Seasonal malaria chemoprevention (SMC) is recommended by the World Health Organization for the sub-Sahel region in sub-Saharan Africa for preventing malaria in children 3 months old to younger than 5 years. Since 2016, the Malian National Malaria Control Program has deployed SMC countrywide during its high malaria transmission season at a rate of 4 monthly cycles annually. The standard SMC regimen includes sulfadoxine-pyrimethamine (SP) plus amodiaquine (AQ). Resistance against SP is suspected to be rising across West Africa; therefore, assessing the effectiveness of an alternative antimalarial drug for SMC is needed to provide a second-line regimen when it is ultimately needed. It is not well understood whether SMC effectively prevents malaria in children aged 5 years or older.

**Objective:**

The primary goal of the study is to compare 2 SMC regimens (SP-AQ and dihydroartemisinin-piperaquine [DHA-PQ]) in preventing uncomplicated *Plasmodium falciparum* malaria in children 3 months to 9 years old. Secondly, we will assess the possible use of DHA-PQ as an alternative SMC drug in areas where resistance to SP or AQ may increase following intensive use.

**Methods:**

The study design is a 3-arm cluster-randomized design comparing the SP-AQ and DHA-PQ arms in 2 age groups (younger than 5 years and 5-9 years) and a control group for children aged 5-9 years. Standard SMC (SP-AQ) for children younger than 5 years was provided to the control arm, while SMC with SP-AQ was delivered to children aged 3 months to 9 years (arm 2), and SMC with DHA-PQ will be implemented in study arm 3 for children up to 9 years of age. The study was performed in Mali’s Koulikoro District, a rural area in southwest Mali with historically high malaria transmission rates. The study’s primary outcome is *P falciparum* incidence for 2 SMC regimens in children up to 9 years of age. Should DHA-PQ provide an acceptable alternative to SP-AQ, a plausible second-line prevention option would be available in the event of SP resistance or drug supply shortages. A significant byproduct of this effort included bolstering district health information systems for rapid identification of severe malaria cases.

**Results:**

The study began on July 1, 2019. Through November 2022, a total of 4556 children 3 months old to younger than 5 years were enrolled. Data collection ended in spring 2023, and the findings are expected to be published later in early 2024.

**Conclusions:**

Routine evaluation of antimalarial drugs is needed to establish appropriate SMC age targets. The study goals here may impact public health policy and provide alternative therapies in the event of drug shortages or resistance.

**Trial Registration:**

ClinicalTrials.gov NCT04149106, https://clinicaltrials.gov/ct2/show/NCT04149106

**International Registered Report Identifier (IRRID):**

DERR1-10.2196/51660

## Introduction

Malaria is endemic to south and central Mali, where over 90% of its approximately 17.6 million population is at risk for infection [1]. The disease primarily burdens rural areas that maintain suitable larval habitats and lack access to adequate health care [1]. Malaria transmission is highly seasonal in Mali (length of the transmission periods varies from 3 to 6 months), with a peak of malaria cases at the end of the rainy season (October through November), though it may be affected by irrigation schemes [2,3]. Deaths due to malaria registered at health centers totaled 1050 in 2017, with 669 (63.7%) occurring among children younger than 5 years. However, these results are likely substantially underreported. For instance, in 2015, health facilities in Mali reported over 2.3 million confirmed malaria cases and 1544 malaria deaths, but actual estimates were 7.5 million and 21,000, respectively [1].

Since 2007, support from the US Presidential Malaria Initiative program and other sponsors resulted in a 50% reduction in Mali’s malaria burden. These efforts have been carried out through increased preventive and treatment measures such as long-lasting insecticide-treated mosquito nets (LLINs), indoor residual spraying, artemisinin-based combination therapies (ACTs), and intermittent preventive treatment of pregnant women (IPTp); and more recently, seasonal malaria chemoprevention (SMC). The most widely implemented malaria control interventions are the joint use of LLINs and rapid treatment of malaria cases with ACTs, IPTp, and SMC. Indoor residual spraying was previously implemented on a small scale in several Malian districts but is currently implemented in only a single district in central Mali.

Despite the broad deployment of these interventions, malaria prevalence and incidence rates remain high in Mali according to the routine Malaria Indicators Surveys [4]. Since 2010, the International Centers of Excellence in Malaria Research (ICEMR), in collaboration with Mali’s National Malaria Control Program (NMCP), has identified significant constraints to malaria control implementation strategies in Mali, including the upward shift of the prevalence of infection and incidence of disease in children younger than 5 years to children aged 5-9 and 10-14 years [5,6]. These findings are particularly significant as the World Health Organization (WHO) guidelines recommend SMC only for children 3 months old to younger than 5 years. Therefore, the Mali NMCP and its partners have expressed SMC effectiveness and intervention strategies as key research priorities.

While the sulfadoxin and pyrimethamine regimen (SP) remains effective in West Africa, resistance to this regimen has been observed in East African regions where *Plasmodium falciparum* is highly prevalent. SMC is not currently recommended for countries in southern and eastern Africa due to widespread resistance, although there are some locations where transmission patterns suggest potential suitability [7,8]. In western parts of Africa, higher frequencies of the triple dihydrofolate reductase mutants and the quadruple mutant (triple dihydrofolate reductase plus dihydropteroate synthetase 437) associated with significant resistance to SP have been observed in children receiving SMC in both Mali and Senegal [9,10]. Recent studies have also reported SP resistance in several parts of Mali [11,12]. The wide-scale deployment of SMC in sub-Saharan African countries required increased focus on *P falciparum* resistance as well as ongoing assessments of new alternative drug combinations for SMC as this strategy has proven to be effective in reducing the impact on severe malaria and mortality in children 3 months old to younger than 5 years [13]. A recent study in Burkina Faso suggests that higher dosages and extended dosing of dihydroartemisinin-piperaquine (DHA-PQ) to 4 monthly doses (and cover the entire high malaria transmission period) could reduce malaria incidence up to 58% during the peak transmission season [14,15]. DHA-PQ has demonstrated excellent efficacy for chemoprevention and benefits from the long half-life of piperaquine and offers a protective efficacy of 98% against malaria in Thai adults when administered monthly [7]. In Senegalese children, similar monthly malaria incidence was observed among children receiving monthly DHA-PQ vs SP+amodiaquine (AQ) as SMC regimen [8].

A study in Uganda reported a 58% greater protective efficacy for DHA-PQ over SP-AQ based on monthly administration among children younger than 5 years [9]. This study aims to determine whether SMC effectively prevents malaria in children aged 5-9 years. The evidence-based approaches used will guide policy in Mali and other countries in West Africa using SMC.

## Methods

### Ethical Considerations

The study was approved by the University of Sciences, Techniques and Technologies Ethics Board under the following reference (N°2019/04/CE/FMPOS). The trial will report the efficacy of 2 SMC subtypes (SP-AQ and DHA-PQ) for preventing *P falciparum* malaria in children aged 5-9 years in Mali. Should the findings show that SMC is efficacious for children in this age group, it may impact policy SMC delivery in Mali.

### Study Site

Mali’s Koulikoro District is situated in its southern region, approximately 50 miles (18 km) north of Bamako and 255 miles (410 km) from the Guinea border [11]. The district maintains 21 health zones and 71 community health posts. The current population in the district is 282,570, with approximately 4% of its population <1 year of age and 18% between 1 and 4 years of age. The total number of villages a community health center covers ranges from 8 to 31. The most populous village is Kolebougou, with 34,712 persons; the least populated is Souban Village, with 5085 persons. Ongoing malaria control activities include case management (rapid diagnostic tests and ACTs), IPTp, SMC, and LLIN use. District health centers maintain clinical and laboratory research capacity and full-time staff and clinicians for malaria screening and patient care. The site was chosen for the proposed research because of its diverse range of malaria control interventions, collaborative research agreements with the University of Sciences, Techniques and Technologies of Bamako, Mali (USTTB), high malaria transmission rates, and rural location.

### Study Design and Population

**Figure 1:**
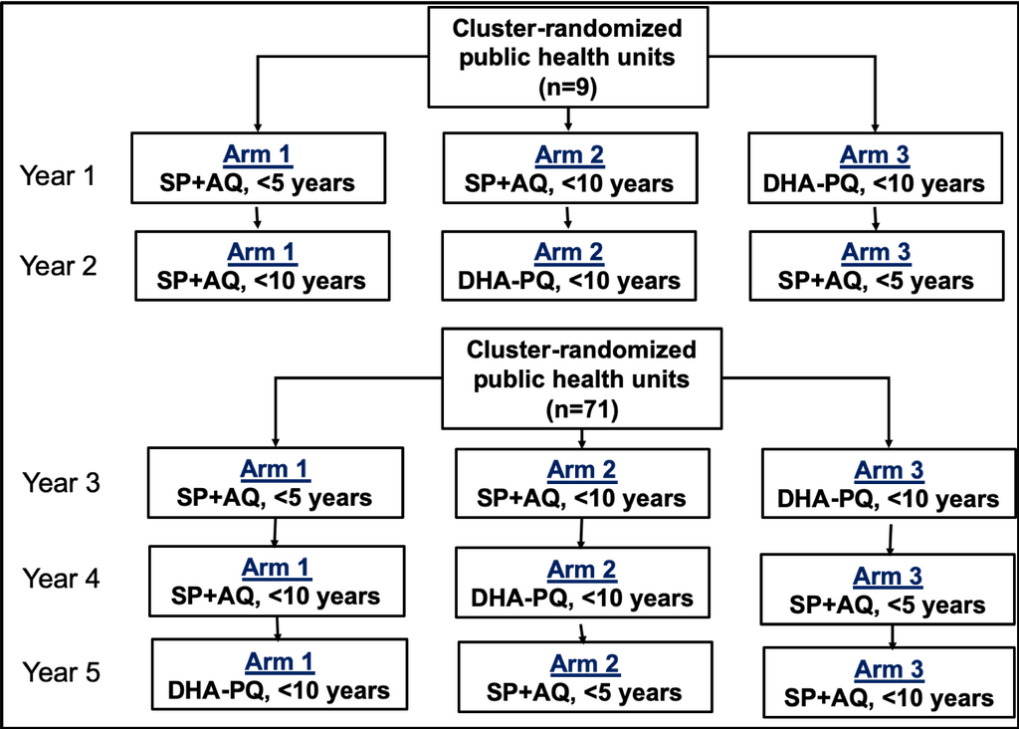
Study design of a 3-arm cluster-randomized trial on age targets for season malaria chemoprevention. The study was performed from July 2019 to March 2023. AQ: amodiaquine; DHA: dihydroartemisinin; PQ: piperaquine; SP: sulfadoxine-pyrimethamine.

The study was as a 3-arm cluster-randomized design, as illustrated in Figure 1.

The study population includes children aged 3 months to 9 years in each village. Each study arm includes 3 corresponding public health units (PHUs) that differ with respect to their ecology, proximity to the Niger River, and region in the context of Koulikoro District (northern, central, and southern).

### Cluster Selection

**Figure 2:**
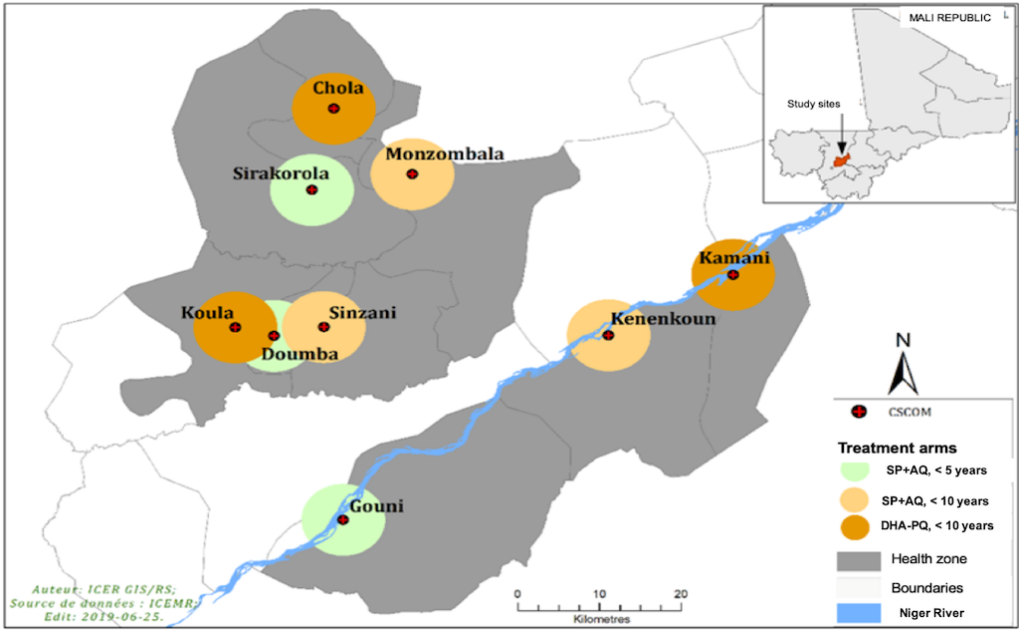
Map of the 9 study sites in Mali’s Koulikoro District. The 9 study sites are near the central part of Mali’s Koulikoro District. Study sites were selected as 3 villages near the Niger River, 3 villages in the northern part of Koulikoro District, and 3 villages in the central part of Koulikoro. AQ: amodiaquine; DHA: dihydroartemisinin; PQ: piperaquine; SP: sulfadoxine-pyrimethamine.

A total of 9 villages were randomly selected from Mali’s Koulikoro District for participation. The selection strategy focused on 3 aspects: proximity to the Niger River (which lies in the southern part of the region) and the central and northern parts of the sampling region. Within these strata, villages were rank-ordered according to their populations. Random selection was carried out such that villages of high, medium, and low populations were sampled from each of these strata. A total of 3 villages (1 village per stratum with different ranking in terms of population size) were assigned to a single treatment arm (Figure 2).

The southernmost villages (Gouni, Kenenkoun, Kamani) are situated near the Niger River, while the remaining villages (Doumba, Sinzani, Koula; and Sirakorola, Monzombala, and Chola) lie in the northern and southern parts of the region above the Niger River, respectively.

### Randomization

Probability proportional to population size sampling was used for allocating villages to the study arm. Random treatment assignment was balanced according to population size in each stratum (low, medium, and high), resulting in a 3 by 3 Latin square arrangement (Table 1).

**Table 1:** Study site classification per sampling region within the Koulikoro District, Mali.

Region	Population size		
	Small	Medium	Large
South	Gouni (A)^a^	Kamani (C)	Kenenkoun (B)
Central	Sinzani (B)	Doumba (A)	Koula (C)
North	Chola (C)	Monzombala (B)	Sirakorola (A)

^a^A, B, and C denote the 3 study arms.

Specifically, PHUs were rank-ordered according to their total populations, and the top 3 PHUs (in terms of their populations) were chosen to represent Koulikoro’s southern, central, and northern regions. The PHUs were then randomized to treatment according to the Latin square arrangement shown in Table 1.

### Eligibility and Enrollment

Participants were eligible to participate if they were residents of the sampled village, aged 3 months to 9 years of age at enrollment, were asymptomatic of current chronic diseases, and did not have a history of allergies to SP, AQ, or DHA-PQ therapies. Informed, written consent is required for all study participants annually. Consent was administered in oral or written formats and included a full description of voluntary participation, the right to withdraw from the study at any time, and the right to refuse to answer any question or participate in any research component.

### Study Outcomes

The primary study outcome is the incidence of severe or uncomplicated *P falciparum* malaria. The population denominator for incidence calculations was derived from the house-to-house enumeration with household member listing. Secondary outcomes were abstracted from censuses and enumeration lists for selected villages, including village-level distributions on populations, age, gender, residential status, and LLIN use. Exhaustive selection was carried out in villages for selected PHUs for those children who met the eligibility criteria and for whom consent of parents or tutors for enrollment was obtained. A baseline malaria infection prevalence survey was carried out before the initial SMC campaign. Follow-up surveys at community health centers were also carried out to assess malaria incidence among study participants. Monthly SMC administration and compliance with treatment assessment were captured via reports by caregivers and measurement of AQ metabolite by enzyme-linked immunosorbent assays. Demographic data, including age, sex, and clinical parameters (including temperature, pulse, and respiration rate), were recorded at a community health center visit. Malaria cases were defined as fever or a history of fever within the past 48 hours associated with either positive malaria rapid diagnostic test or a positive blood smear prepared during that visit.

### Hypotheses and Rationale

The primary hypotheses were as follows:

1. Malaria incidence in children was at least 10% higher in the 5- to 9-year age group without SP-AQ than in children in the 5- to 9-year age group with SP-AQ.
2. Malaria incidence in children 3 months old to younger than 5 years receiving DHA-PQ was not statistically different from children in the same age group receiving SP-AQ.

### Data Collection and Management

Confirmed malaria case incidence data will be collected from public health facilities through electronic data capture. A REDCap (Research Electronic Data Capture; Vanderbilt University) database and mobile app were used for this study, and data synchronization will be performed daily for quality checking complemented with data queries distributed to the centralized data center at USTTB.

### A Priori Sample Size and Statistical Power

The power and sample size assessment is based on the ability to detect a clinically significant difference in malaria incidence proportions between the 3 comparison groups in year 1. The minimum clinically meaningful differences between the comparison groups was set at 10%. The sample sizes were calculated assuming a design effect of 2 with a 2-sided type I error set at 0.05 and power set at 80%. The number of required subjects per study arm for detecting at least 10% differences in malaria incidence proportions was 1552 in year 1. This result was inflated by 233 subjects in year 2 to account for new subjects due to increased child ages, yielding 1785 participants per arm total or 5355 participants overall.

### Phase 2 (Years 3-5, 2021-2023)

**Figure 3:**
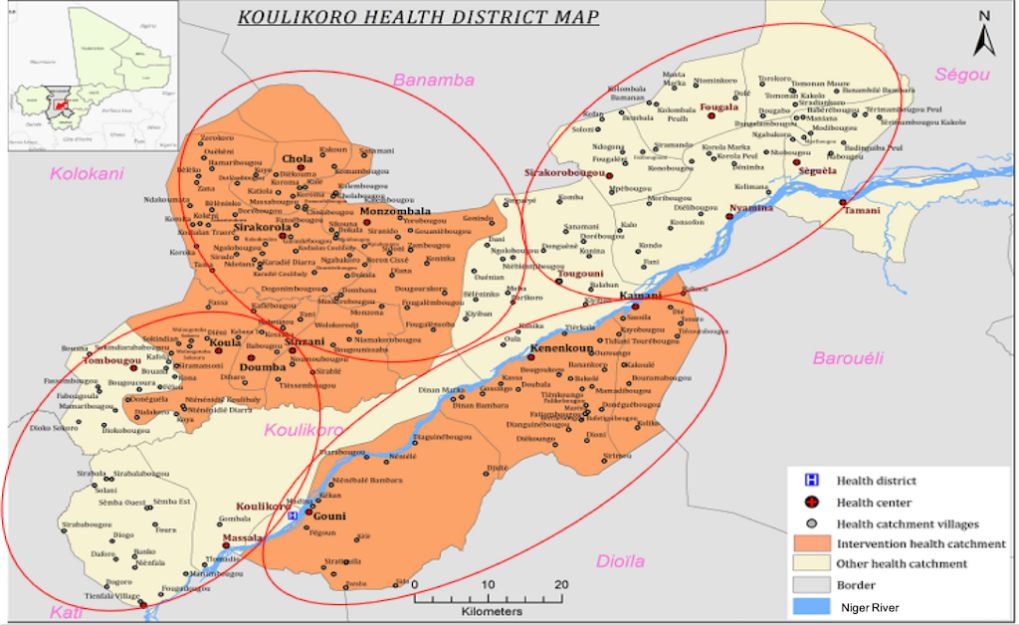
Map of the selection approach for 71 public health posts in Mali’s Koulikoro District.

This phase involved expansion to 71 public health posts in years 3-5 (Figure 3). The district-wide data collection plan will build on the efforts for years 1-2 and focus on (1) cluster-randomized health posts to the 3 treatment arms and (2) semiannual community cross-sectional surveys. More specifically, the 71 health posts over the entire district were divided into 4 regions (Table 2).

**Table 2:** Stratum matched by the year of the study.

Stratum (region), years 3-5 sampling approach	Stratum (region), years 1-2 sampling approach
Northeast	Not done
Northwest	North
Southwest	Central
Southeast	South

Within the stratum, health posts will be randomized to 1 of 3 treatment arms (SP-AQ <5 years, SP-AQ <10 years, and DHA-PQ <10 years). Allocation was proportional to population size based on census populations up to 9 years of age while balancing on population sizes across the 3 study arms. For regions maintaining a total number of health posts that were not divisible by 3, 1 or 2 adjacent health posts were considered as a single unit to ensure divisibility (Figure 3).

The health facilities selected in years 1 and 2 were also be selected in years 3-5 and will leverage the process and training already in place in 9 health centers. Patterns over the entire 5-year study period will be analyzed for these 9 villages. Semiannual cross-sectional household surveys will permit assessment of community-level effects over the entire 5-year period.

## Results

The study began on July 1, 2019. Through November 2022, a total of 4556 children were enrolled during the pilot phase (2019-2020) in 9 villages across the Koulikoro Health District. In 2022, preliminary findings have been presented at the American Society of Tropical Medicine Conference [16] and published in the *American Journal of Tropical Medicine and Hygiene* [17]. SP-AQ and DHA-PQ were highly effective in reducing *P falciparum* malaria in children 5-9 years in Koulikoro, Mali, at both the pilot and district-wide study phases. Data collection ended in spring 2023, and the findings are expected to be published later in early 2024. Results will be summarized and reported using both intention-to-treat and per-protocol analyses.

## Discussion

This study was designed to assess the effectiveness and efficacy of SP-AQ in children aged up to 9 years and of DHA-PQ as an alternative in the event of drug resistance to AQ or SP in West Africa and, more broadly, across Africa. The trial included 2 SMC regimens to provide a viable alternative therapy in the event of drug resistance or shortages in drug supply. The study enrolled over 5000 subjects in 9 villages in Mali’s Koulikoro District. The expanded phase of the study covered the entire Koulikoro District. The initial phase of the study covered 9 villages, which may be considered as a pilot for the larger district-wide trial.

This study provides an opportunity to directly measure the effect of the 2 drugs while extending SMC to older children in response to recent reports showing an age shift in malaria incidence and prevalence among older children. The study site here comprised different ecological settings represented countrywide with differential malaria transmission intensities. For instance, each study arm was composed of a village located along the Niger River where longer transmission seasons have historically occurred, a village located in the central part of Koulikoro District where malaria transmission peaks between July and October, and a village in the southern part of Koulikoro District where malaria transmission season has been historically shorter (August to November) than the other 2 locations. Also, all selected villages maintain community health facilities where malaria case management is done routinely. Additionally, a routine assessment of treatment compliance by measuring amodiaquine metabolite in the veinous blood of children after treatment will be performed for the first time to determine SMC compliance across all 4 dosing periods. While the number of villages was initially restricted to 3 per study arm, all 71 PHUs in Koulikoro will be covered across the 3 study arms should the study meet the go/no go criteria for continued funding.

Particular challenges at the outset of the study planning involved garnering support from regional health administrators about using DHA-PQ instead of SMC and extending SMC use to children aged 5-9 years. Since the inception of Mali’s NMCP, a key partner in this effort made apparent the study’s rationale in terms of its implications on potential drug resistance with health agents, community leaders, and health workers before the SMC campaign was launched in Mali. Determining the appropriate age target for preventive therapies remains challenging for developing countries like Mali. The age captured during the most recent census was used to estimate the number of children failing in the <5 and 5-9-year age groups, which was usually reported by the participants’ parents or guardians. However, approximately 2% of subjects were misclassified during the SMC administration due to erroneous reporting during the census. Therefore, more accurate estimations of participant age (which was used to define the comparison groups) were captured for the study here at baseline.

SMC covers the rainy season that coincidentally occurs during school vacations and agricultural activities in rural Mali. Therefore, a child’s movement during that period is often a cause for losses to follow-up as children frequently might travel to Mali’s capital city of Bamako from July to September, while others will move with their family to the farms (hamlets) until October when school commences.

We believe that this study provides a somewhat novel study design that will aid researchers in assessing age targets in locations where controlled studies are not feasible.

## Appendix

Multimedia Appendix 1 Peer review report from the National Institute of Allergy and Infectious Diseases Special Emphasis Panel Limited Competition: Revision Applications for International Centers of Excellence for Malaria Research (NIH, USA).

## Abbreviations

ACT: artemisinin-based combination therapy
AQ: amodiaquine
DHA: dihydroartemisinin
ICEMR: International Centers of Excellence in Malaria Research
IPTp: intermittent preventive treatment of pregnant women
LLIN: long-lasting insecticide-treated mosquito net
NMCP: National Malaria Control Program
PHU: public health unit
PQ: piperaquine
REDCap: Research Electronic Data Capture
SMC: seasonal malaria chemoprevention
SP: sulfadoxine-pyrimethamine
USTTB: University of Sciences, Techniques and Technologies of Bamako, Mali
WHO: World Health Organization

## Data Availability

Data will be archived on the ClinEpiDB institutional repository [18] to ensure long-term availability, preservation, and access. The data sets generated during this study are available from the corresponding author on reasonable request as well as from the West Africa International Centers of Excellence in Malaria Research (ICEMR) Program Director by e-mail (sdoumbi@icermali.org or sdoumbia@gmail.com)
