# Supplementary material for: Seasonal Malaria Chemoprevention Therapy in Children Up To 9 Years of Age: Protocol for a Cluster-Randomized Trial Study
Source: JMIR Res Protoc. 2024 Jan 22;13:e51660. doi: 10.2196/51660 (PMC10845024; doi:10.2196/51660)
Supplement: Multimedia Appendix 1 [file resprot_v13i1e51660_app1.pdf]

SUMMARY STATEMENT
(Privileged Communication)

PROGRAM CONTACT:
Malla Rao
240-627-3352
mrao@niaid.nih.gov

Release Date: **10/12/2018**
Revised Date:

Application Number: **3 U19 AI129387-03S1**

Principal Investigator

DOUMBIA, SEYDOU

Applicant Organization: UNIV OF SCIENCES, TECH & TECH OF BAMAKO

Review Group: **ZAI1 AMC-M (J1)**
National Institute of Allergy and Infectious Diseases Special Emphasis Panel
Limited Competition: Revision Applications for International Centers of Excellence for Malaria Research (U19 Clinical Trial Optional)

Meeting Date: **09/26/2018**
Council: **JAN 2019**
Requested Start: **04/01/2019**
RFA/PA: **AI17-044**
PCC: **M90**

Project Title: **ICEMR-Mali : Multidisciplinary research for malaria control and prevention in West Africa**
SRG Action: **Impact Score:38**
Next Steps: **Visit https://grants.nih.gov/grants/next_steps.htm**
Human Subjects: **30-Human subjects involved - Certified, no SRG concerns**
Animal Subjects: **10-No live vertebrate animals involved for competing appl.**
Gender: **1A-Both genders, scientifically acceptable**
Minority: **1A-Minorities and non-minorities, scientifically acceptable**
Children: **1A-Both Children and Adults, scientifically acceptable**

[redacted]

ADMINISTRATIVE BUDGET NOTE: The budget shown is the requested budget and has not been adjusted to reflect any recommendations made by reviewers. If an award is planned, the costs will be calculated by Institute grants management staff based on the recommendations outlined below in the COMMITTEE BUDGET RECOMMENDATIONS section.

FOREIGN INSTITUTION

RESUME AND SUMMARY OF DISCUSSION: This excellent application entitled “ICEMR-Mali : Multidisciplinary research for malaria control and prevention in West Africa” was submitted in response to RFA-AI-17-044, “Limited Competition: Revision Applications for International Centers of Excellence for Malaria Research (U19 Clinical Trial Optional)” by the University of Sciences, Techniques and Technologies of Bamako, with Dr. Seydou Doumbia as Principal Investigator. The overall goal of this supplement is to improve the effectiveness of implementation of Seasonal Malaria Chemoprevention (SMC) and referral and management of severe disease, to reduce the burden of malaria in rural Mali and similar settings.

Two projects are proposed, led by Dr. Petter Winch (Project 1) and Dr. Mahamoudou Toure (Project 2). Project 1, “Research for effective implementation of SMC strategy, and pre- and post-referral management of severe malaria in Mali,” will examine social, cultural, economic and health systems factors associated with effective implementation of Seasonal Malaria Chemoprevention (SMC) and pre-referral treatment followed by immediate referral of children with severe disease in the community to health facilities. Project 2, “Effectiveness of Seasonal Malaria Chemoprevention in Koulikoro, Mali” will evaluate the effectiveness of SMC in Mali with respect to strategy implementation, cost and efficacy of SMC on reducing burden of malaria in children up to 10 years of age and the potential impact of drug resistance. Projects 1 and 2 scored in the Very Good range.

This program includes well planned studies addressing important issues in malaria control, and could contribute to a better control of malaria in Mali through effective implementation of SMC. The rationale for the proposed studies is supported by data from national malaria control program and partners indicating suboptimal implementation of the SMC and severe malaria interventions, and the studies were initiated in response to priorities set by the program and its implementing partners. The proposed study to test the hypothesis that the effectiveness of SMC will depend on the implementation, environmental factors, extent of drug resistance, and the presence of mosquito vector is timely and significant. Sites and focus of studies are well-considered and justified by epidemiology and current data on intervention coverage and performance, and collaborations across institutions and other ICEMRs are evident. The studies proposed are led by a proven team of researchers in Mali and at global partner institutions with a long history of collaboration on previous malaria research ranging from biomedical to social and behavioral aspects of interventions for prevention and control. Trial design and approach are well conceived and potentially able to produce robust outcomes that could affect expansion of policy. Existing data from malaria immunology studies and the prevalence of antimalarial drug resistance markers will be informative for the proposed work. There are a few weaknesses which reduce enthusiasm for the overall application. The focus of the proposed observational studies is well-justified and sound, but will require additional data collection designs and tools; the parent award’s baseline data from its electronic data capture investment for passive case detection data at health facilities won’t be sufficient for either of the proposed studies. The trial design is insufficiently specific, clear or detailed to assess its likelihood of success with confidence. The application could benefit from additional coordination between clinical researchers and biostatisticians.

Overall, while the application almost certainly will yield information that can improve the delivery of two currently recommended interventions in Mali, it is unlikely to build or advance the field of antimalarial interventions overall. Based upon the evaluation of scientific and technical merits of the projects and cores and the interactions among them in the overall Program, this Program Project received an Overall Impact score of 38.

[redacted]

Project 2:
Title: - Effectiveness of Seasonal Malaria Chemoprevention in Koulikoro, Mali
Project Leader: Toure, M

Project 2 proposes to evaluate the effectiveness of SMC in Mali with respect to strategy implementation, cost and efficacy of SMC on reducing burden of malaria in children up to 10 years of age and the potential impact of drug resistance. The specific aims are to (1) Investigate the effectiveness of SMC in four health zones in Mali's Koulikoro District, (2) Determine the financial cost per malaria case averted and per child dosed by age group (<5, 5-9 yrs.) in Mali's Koulikoro District and (3) Determine effectiveness of SMC on children by age group (<5, 5-9 years) over Mali's entire Koulikoro District using a household randomized controlled trial study design.

The sequence of studies proposed is well designed to achieve a good understanding of the underlying issues that affect effectiveness of SMC. Aim 3 cluster randomized trials in particular have the potential to be definitive trials with regard to efficacy of SMC. The team is strong and possesses the expertise needed to complete this work, including a demonstrated ability and experience in conducting trials in this setting. The sequence of trials is sensible and well described, and outcomes have been clearly defined. The project includes innovative ELISA assay development and implementation. Sample size has been planned with attention to statistical considerations, which generates confidence to scientific rigor. Facilities and resources are excellent for successful completion of the research proposed. There are several major weaknesses which decrease enthusiasm for the project, however. A concern is that the districts may not meet the conditions suitable for SMC intervention; they are described as having "virtually year-round malaria transmission," so the reason for lower effectiveness from the seasonal approach may be the lack of seasonality. This possibility was not discussed. The evaluations and hypotheses planned for the cohort and cross-sectional study are unclear and confusing. The primary exposure variable (SMC exposed and non-SMC-exposed children) is not defined, given that all communities are receiving SMC as the standard of care. Evaluation of whether child age is an important factor in SMC implementation may be studied, but this is not clear. The use of the two different age group exposures to SMC in the 4 communities is not explained, and there are discrepancies between the approach and stated hypotheses and the sample size/analysis sections in what the primary assessments goals are. The Go/No Go criteria are not appropriate given the requirements of the RFA; rather than a Go/No Go, the criteria present an alternative approach depending on the results of the first two years of the study.

Overall, the trials proposed have the potential to generate important information regarding SMC, but additional clarity is needed for several aspects of the approach. Based upon the evaluation of scientific and technical merit, Project 2 received an Overall Impact score of 49.

CRITIQUE: The comments in the CRITIQUE section were prepared by the reviewers assigned to this application and are provided without significant modification or editing by staff. They are included to indicate the range of comments made during the discussion, and may not reflect the final outcome. The

DESCRIPTION (provided by applicant):
Despite wide deployment of malaria control interventions in the past decade, Sub-Saharan Africa continues to carry a disproportionate share of the global burden and of malaria-related deaths. Two key strategies for reducing the malaria burden in young children are Seasonal Malaria Chemoprevention (SMC), and pre- referral treatment, followed by immediate referral of children with severe malaria in the community to health facilities equipped for appropriate management of life-threatening malaria. However, despite significant investments in malaria control interventions by the Government of Mali and donors, the impact on burden of the disease in young children has been less than expected. Thus, the overall goal of this application is to improve the effectiveness of 1) SMC implementation and 2) Referral and management of severe disease in rural Mali and consist of two projects: a Study Type 2 (Observational studies) and Study Type 3 (Implementation studies). The specific aims of Study Type 2 are: 1) To describe the community and health systems context for implementation of SMC, and implementation of protocols for referral and management of severe malaria; 2) To explore factors determining success, and barriers and pitfalls related to the SMC implementation strategy including perception of SMC in the population and by health providers, delivery strategy and coverage, and sustainability; and 3) To evaluate household and health system responses to severe malaria, including provision of appropriate pre-referral management, and implementation of case management protocols. These aims will be addressed through quantitative and qualitative surveys and observations at community and health facilities levels. The Study Type 3 will assess the effectiveness and long-term

impact of SMC on the epidemiology of malaria. The specific aims are: 1) To assess the impact of extended SMC on the incidence and prevalence of malaria among children under 5 and older children (5-10years) in intervention areas vs. control; 2) To determine the long-term effect of SMC on acquisition of immunity, parasite population structure, and drug resistance markers 3) To assess the cost – effectiveness of SMC delivery and implementation strategies. The project will provide an essential evidence basis for improving these malaria control interventions in Mali and in West Africa.

PUBLIC HEALTH RELEVANCE
Malaria remains a major life threatening disease across sub-Sahara Africa, particularly among children less than 5 years of age and pregnant women. Because malaria control interventions are being focused on children under 5 years old in many countries, the burden of the disease is shifting on older children. This field-based research will contribute not only to provide plausible evidence of the effectiveness of key malaria control intervention, but also determine a potential long-term impact of the malaria control strategies on the epidemiology of the disease.

Project-001 - Research for effective implementation of SMC strategy, and pre- and post-referral management of severe malaria in Mali

DESCRIPTION (provided by applicant): Two key strategies for reducing malaria-related mortality in young children are Seasonal Malaria Chemoprevention (SMC), and pre-referral treatment followed by immediate referral of children with severe disease in the community to health facilities equipped for comprehensive management of these children. This suggested study will examine social, cultural, economic and health systems factors associated with effective implementation of these two strategies. The three specific aims are to: 1) To describe the community and health systems context for implementation of SMC, and implementation of protocols for referral and management of severe malaria; 2) To explore factors determining success, and barriers and pitfalls related to the SMC implementation strategy including perception of SMC in the population and by health providers, delivery strategy and coverage, and sustainability; and 3) To evaluate household and health system responses to severe malaria, including provision of appropriate pre-referral management, and implementation of case management protocols. These aims will be achieved through quantitative and qualitative interviews with health officials, community and facility-based health care providers, and parents of young children. We will carry out a survey of markets and ambulatory drug sellers and evaluate training, work and supervision of SMC distributors. Furthermore, we will conduct follow-up interviews and observation of parents on days 2 and 3 after SMC distribution, and a household survey to estimate coverage at end of SMC distribution, and measure factors affecting effective implementation of SMC. A village-based surveillance system to identify cases of severe disease will be established in the two villages, accompanied by interviews with parents, providers and anyone else coming in contact with the child. The cause of severe illness will be classified with a standard WHO algorithm; and social, economic, cultural and health system causes with the BASICS Pathway to Survival tool. Lastly, we will monitor the management of severe malaria in community health centers and district hospital. The expected outcomes of this work, upon completion of our specific aims, include 1) Recommendations to Malian health officials and other partners for improving implementation of SMC and referral and management of severe malaria, and 2) Guidelines for routine monitoring of SMC implementation and development of mechanisms for responses to problems as they occur.

CRITIQUE 1

Significance:	5
Investigator(s):	2

Innovation:	6
Approach:	2
Environment:	3

Overall Impact:

The proposed project provides a detailed response to a need for operations research to improve the effectiveness of malaria control interventions that was developed in collaboration with the national malaria control program and its development partners. If funded and completed this project is likely to yield findings that will incrementally improve two specific malaria control interventions that are already widely recommended and considered standard of care in Mali. This could result in meaningful improvements in malaria control and child survival. It is also possible that the study would reveal system bottle necks that constrain the effectiveness of SMC and severe malaria case management in other malaria-endemic countries (The investigators might wish to consider developing a streamlined approach for similar assessments that can be undertaken by program implementers without an extensive research infrastructure as an additional product of this work). However, it is hard to imagine that the project would exert a sustained, powerful influence on the research field. That is not its intent.

1. Significance:

Strengths

- The project proposes to address critical community and health systems factors that constrain two widely recommended malaria control interventions.
- It has been developed in response to (and in collaboration with) a specific concern of the national malaria control program and its development partners.
- It is justified through research and program data indicating incomplete implementation of the two interventions.
- If implemented as described the project is very likely to create information that will improve the effective implementation of SMC and comprehensive management of severe illness which could reduce the burden of malaria and prevent childhood deaths in Mali and potentially other countries with similar malaria burden, epidemiology and policy context.
- The proposed methodology is comprehensive and appropriate to assessing interventions in a complex community and health systems context. The implementation research framework is well suited to the issues under investigation.

Weaknesses

- By addressing currently recommended malaria interventions, the project is unlikely to advance the field considerably.
- Improving SMC and comprehensive management of severe disease are both likely to improve child survival only marginally and can be expected to have very limited impact on malaria transmission.
- The currently described study does not include any measure of human health impact nor a plan to evaluate the impact of changes made as a result of the planned studies.
- The proposed methodology is too complex to be a model for other program managers to adopt as a means of assessing and overcoming barriers as part of routine program implementation.

2. Investigators:

Strengths

- Both named investigators at USTTB and JHU are experienced social science and public health researchers who have contributed to the development and evaluation of public health program interventions at community and health systems level. Levels of training and prior collaboration records suggest that they are both ideally positioned to support this work.
- There is good evidence that malaria control officials and partners are supportive of the work and keenly interested in the findings.

Weaknesses

- Level of effort for each of the named investigators is limited to <15%, meaning that most of the responsibility for completing the work as planned will have to rest with staff yet to be identified and who may have only occasional contact with the lead investigators.
- Coinvestigators in the national malaria control program or regional/ district health authorities are not named or proposed. The role and level of effort for the USG co-investigator are not clear. Investigators and program managers should be aware of the potential for institutional barriers that might limit the participation of USAID staff.
- There is a lack of health economics expertise among the named investigators which may need to be addressed given that economic influences and barriers are described in the primary goal and specific objectives alongside the social, cultural and health systems ones.

3. Innovation:

Strengths

- The proposed approach, while not innovative, could be framed as a starting point for developing a more manageable set of tools that district and regional health authorities might be able to employ to assess an improve the operations of their programs in real world settings.
- The application aims to refine and improve recommended interventions that are currently under performing and could result in reorienting them.
- It might also reveal information about the performance of malaria control tools that is valuable in the interpretation of the ICEMR-Mali's other immune-genomic and transmission studies.

Weaknesses

- Innovation is not the point of this particular project. The interventions under investigation are already standard of care (though inconsistently implemented); the implementation science approach is well established in the malaria control community (despite claims otherwise in the application); the conceptual framework/ theory of change at the heart of this application is likewise familiar; and the mix of qualitative and quantitative assessment tools is not unusual. That should not detract disproportionately from the fact that this is an opportunity to bring these together in a carefully planned and examined way that is often not possible once interventions graduate beyond the trial phase.
- This may be better suited to support from the US President's Malaria Initiative. It is clear that staff from the USAID mission have been involved in developing and intend to participate in this project. Why wouldn't this work be supported under their portfolio?

4. Approach:

Strengths

- The investigators propose a rich mix of qualitative and quantitative data collection activities including both observations and interviews targeting community members, child caretakers, community health agents and volunteers, drug sellers, SMC distributors, facility-based health workers, public health program managers and their partners—all of which should provide a comprehensive perspective on the context in which SMC and management of severe malaria illness are intended to be delivered.
- A quantitative (and, presumably, representatively sampled) household survey will be used to estimate SMC coverage at the community level.
- The application has appropriately adapted the methods to accommodate the broad experience that consumers and health system actors have already had with SMC as well as the lack of familiarity with some of the severe malaria commodities and approaches.
- Characterizing severe illness according to WHO classifications and BASICS Pathway to Child Survival will provide an important point of reference.

Weaknesses

- Most of the data collection described is qualitative in nature and unlikely to be collected in a way that is intended to produce population based statistics. There is little detail provided on how communities will be selected, and even less about the considerations that will guide recruitment and enrollment of individual participants.
- Budgets are provided for qualitative and quantitative analysis software, but details of how data will be collected, transformed and analyzed are not provided…nor even generally described.
- The inventory of drug seller's stocks and sampling of available medicines is not adequately justified. How will this information be used? What is the intention of collecting sample medications?
- Managing, reducing, storing and manipulating such a wide range of largely qualitative and text-based data may present challenges that are unfamiliar to the clinical and laboratory orientation of the ICEMR-Mali's data management personnel and facilities. What measures will be taken to prepare them for this.

5. Environment:

Strengths

- Without question, the scientific environment in which these studies are to be conducted will enhance the proposed work. The availability of additional research resources and infrastructure, should improve the quality of work being done. There is the potential for findings from this implementation science to inform (and be informed by) the other epidemiological, immune-genomic and transmission research ongoing at ICEMR-Mali.
- The environment is also strengthened by the strong policy commitment on behalf of the national malaria control program and its partners to the SMC and severe malaria interventions and their express interest in facilitating and learning from this proposed work.

- Conducting these investigations in field sites that are well-characterized can allow the investigators to explore many more potential associations than would have been possible with a more conventional approach to an operational research agenda.
- Letters from malaria program and US PMI document commitment to providing resources to ensure high level coverage of interventions under investigation.

Weaknesses

- Potential links to the broader ICEMR-Mali investigations and field sites are not described at all. The application almost seems to turn its back to the rest of the ICEMR work and agenda.
- Conversely, the association with the larger research agenda and use of the same, overlapping or contiguous field sites can have important implications for implementation science. If the setting is too altered by the research community presence, it is very likely that the situation will not be typical of other parts of the country. Frequently such settings are relatively better served than is typical (such as the long history of IRS). Health systems and communities will have adapted to researchers' presence in ways that distort the findings.

Study Timeline: ACCEPTABLE.

ADDITIONAL COMMENTS TO APPLICANT:
Comments:

- The investigators may wish to consider how to package their experience and tools for examining context and influences on effective implementation of both SMC and comprehensive management of severe illness into a package that district- or sub-national level health officials and malaria focal persons could use to assess their own situations and make improvements in delivery of their programs
- The nature of this work seems to be largely operational and may be suited to support from the US President's Malaria Initiative, given their expressed commitment to systematically investigating and overcoming barriers to effective implementation of the proven malaria control tools they support countries to implement and expected contribution to implementation of SMC in Mali. It's noteworthy that PMI has participated in developing this application. Wouldn't they value funding it directly?

CRITIQUE 2

Significance:	3
Investigator(s):	4
Innovation:	3
Approach:	2
Environment:	3

Overall Impact:

The Investigators propose to study two very important strategies used in malaria control – Seasonal Malaria Chemoprevention and Pre-referral treatment for Severe Malaria using Rectal Artesunate. The proposed study builds on a previous formative research, evaluation of implementation of SMC and community acceptance of pre-referral treatment in the same setting. They plan to carry out some behavioral studies in order to understand clearly what the facilitators and barriers to effective implementation of both strategies are. The project has been generally well described and detailed.

Though the studies proposed are not innovative, they can potentially provide some insight into critical issues relating to the development of immunity and the shift in the age group that is vulnerable to malaria. There will be a need to strengthen capacity building and some ethical issues.

1. Significance:

Strengths

- The study is being carried out in an area which is ranked 5th highest in the world for U5 mortality rate in 2016 with malaria accounting for a large proportion of the deaths
- The project seeks to address an issue that is very important to Mali - seasonal malaria chemoprevention (SMC) and pre-referral treatment of severe malaria. Though recognized as a very effective intervention, there appears to be challenges with its implementation, with coverage being less than expected.
- The Investigators plan to carry out some observational studies to explain what they see as an SMC implementation problem

Weaknesses

- The issue of pre-referral treatment of severe malaria and the implementation challenges it poses currently in Mali, though important may be of moderate significance and potentially more relevant to the local setting and not generalizable as this may be as a result of a number of context issues

2. Investigators:

Strengths

- Samba Diop is a Professor in Human Ecology, Anthropology and Ethics Studies at the Department of Public Health and Faculty of Medicine and Dentistry. He has experience in community involvement studies, community behavior change intervention, observational studies and bio ethical studies on malaria, HIV, EBOLA and other infection diseases in Mali and other African countries
- Prof Peter Winch Professor in the Social and Behavioral Interventions (SBI) Program in the Department of International Health, Johns Hopkins Bloomberg School of Public Health with experience in global health, qualitative and formative research, design of behavior change interventions, and applied medical anthropology.
- The third person is the USAID/PMI Resident Advisor, Dr. Mihigo, not that widely published.

Weaknesses

- While there is no doubt about the research experience and skills of the Project Leader who has over 20 years of research experience, and other members of the team, his research interests have not been that focused on the area they are proposing to work in and have over the years included a variety of areas such as - climate change adaptation, water and sanitation, maternal and newborn interventions, community health worker, to mention a few.
- Only three out of all the 16 team members listed for the entire project and two of the several collaborating Institutions are dedicated to this study

- Personnel to carry out the huge amount of work the proposed study entails is of concern. There are no named middle level personnel in the team and one of the three members may not really be involved in the day to day running of the research.

3. Innovation:

Strengths

- The results of the study are likely to contribute directly to malaria program improvement specifically with regard to these two strategies for malaria control

Weaknesses

- The project and methods proposed as described in the application themselves are not novel.

4. Approach:

Strengths

- The overall approach of including behavioral research to identify the best strategies for effectively implementing SMC and Pre referral treatment of malaria effectively is good as it will provide information to direct the design of the subsequent implementation studies
- The studies are being carried out in areas where the parent ICEMR carried out earlier work thus capitalizing on historical data and established systems for the proposed work.
- The three specific aims are well elaborated and clear
- The application proposes to make use of a range of methods which will provide a lot of rich relevant data that will provide information for improvement of the program including:
    - qualitative interviews with NMCP and NGO staff, district and regional health officials; quantitative interviews with healthcare providers, community health workers, a survey of markets and ambulatory drug sellers, collect samples of antimalarial
    - examination of the training, work and distribution of SMC distributors, interviews with the distributors and their supervisors, follow-up interviews with parents, and household surveys to estimate coverage and measure factors affecting effective implementation
    - setting up of a village based surveillance system in 2 villages to identify cases of severe diseases while conducting qualitative and quantitative interviews with caretakers, community health workers, facility-based providers and examine how severe malaria is managed in health facility
- The Investigators have long-term long standing collaborations with several external Institutions

Weaknesses

- The expected outcomes as stated are however a bit weak and disappointing. The application refers to outcomes that include recommendations to health officials, guidelines for routine monitoring of SMC and an understanding of the causes of implementation problems.

- The Team appears to be expanding the scope of the work to studies whose results can feed directly into the control program and improvements in its operations. Capacity Building however, which will contribute to building up the system in a sustainable manner is either missing component in the proposed project or not well outlined.

5. Environment:

Strengths

- The Investigators appear to have established close links with the NMCP and PMI leading to discussions of research priorities to support malaria control in Mali

Weaknesses

- The linkage with the health system and the districts where the proposed work is to be done does not appear to be that strong as no letter of support from them was submitted.

Study Timeline: ACCEPTABLE

CRITIQUE 3

Significance:	2
Investigator(s):	1
Innovation:	2
Approach:	1
Environment:	1

Overall Impact:

Overall a very strong application, which with only a few exceptions, asks all the most important questions about SMC and seeks to address them constructively but nevertheless thoroughly and objectively. Methodologically convincing and proposed by an outstanding team of investigators with a proven, exemplary strategic vision for institutionalization and development in Mali.

1. Significance:

Strengths

- Regardless of the outcome, the proposed study is of very high significance. If SMC has a sustainable future in malaria prevention, that's a very big win but will clearly come at a substantial cost. However, even if, as I suspect, it doesn't turn out to be as exciting an option as previously thought, it's important to know that and also to know that all avenues for maximizing effectiveness have been rigorously explored.
- The emphasis of the operational research components is well-grounded in a solid understanding of practical realities of implementation in Mali, and largely asks all the right questions about bridging the gap between efficacy and effectiveness. The practical, applied and pragmatic perspective is really impressive and based on impressively detailed awareness of the issues on the ground.

- The emphasis upon cost is really an important component and seeks to answer perhaps the toughest question that needs to be asked about SMC.

Weaknesses

- Some of the introductory referencing is years out of date and conveys a worrying unawareness of the most current, authoritative literature.

2. Investigators:

Strengths

- An outstanding set of PIs with a long history of working together.
- The way in which the PIs have transitioned their roles over the years to really center the leadership of this initiative in Mali is laudable, and exactly what I'd like to see from all ICEMR consortia.

Weaknesses

- None.

3. Innovation:

Strengths

- Really focused on practical applied issue relating to effective delivery and satisfactory impact from what is a relatively new and innovative malaria prevention strategy.
- Drawing upon experiences with TB therapy brings exactly the kind of fusion and critical mass such large centers of excellence are intended to deliver, and makes the best of existing capacities at USTTB.
- The genomics content is important and adds value to the study in terms of insight.

Weaknesses

- None

4. Approach:

Strengths

- Largely very strong, convincing and detailed methodology.
- The objective, even-handed perspective and emphasis upon clear definitions of processes and outcomes are considerable strengths.

Weaknesses

- My only substantive concern relates to the focus on personal protection amongst targeted age groups, which is more a limitation in the rational and significance than the approach per se.

- I'd like to see more detail and references about how the sample size calculations were accomplished. As written, the reader is expected to take these somewhat vague descriptions on trust.

5. Environment:

Strengths

- A really outstanding environment in terms of not only consortium-wide expertise but also local infrastructure and human capacity in Mali
- Proven track record of commitment and success with institutionalizing capacity at the national institution which now leads the overall effort.

Weaknesses

- None

Study Timeline: ACCEPTABLE

ADDITIONAL COMMENTS TO APPLICANT:
Comments:

- In future applications I would recommend explaining more clearly (1) Why older children are harder to reach with interventions
- In order to improve the sustainability and independence of USTTB, I would recommend transition from the sundry proprietary statistical packages they currently use to R open source software.

Project-002 - Effectiveness of Seasonal Malaria Chemoprevention in Koulikoro, Mali (Description as provided by applicant)

DESCRIPTION (provided by applicant): Seasonal malaria chemoprevention (SMC), is a prophylactic antimalarial regimen of sulfadoxine- pyrimethamine (SP) and amodiaquine (AQ) recommended by the World Health Organization (WHO) for pre- venting malaria episodes in children under 5 years of age in specific, highly seasonal, transmission settings. In 2012, the WHO called on all countries in the Sahel sub-region of Africa to implement SMC. The WHO reports that SMC is 75% effective in preventing all malaria episodes and 75% of severe malaria episodes when implemented according to its guidelines. From 2013-2016, the West African International Centers of Excellence for Malaria Research (ICEMR) carried out a cohort study including 1,814 subjects in a remote village in Dangassa, Mali. Peak malaria incidence reductions following SMC coverage in children less than 5 years of age were observed in 2015 at 38%, falling far short of the 75% protective efficacy suggested by the WHO. These estimates call into question the effectiveness of SMC implementation or the efficacy of the therapy itself in high malaria transmission in Mali like Dangassa. The purpose of the proposed study is two-fold. First, cross-sectional and cohort studies are used to establish levels of SMC coverage and implementation practices to assess effectiveness of SMC. Second, if results suggest operational, implementation, SP or AQ resistance and SMC coverage practices are acceptable, a randomized control trial will be conducted to measure the effective- ness of extending SMC to children up to 10 years of age. Alternatively, if operational and implementation strategies are found to be functioning poorly and drug resistance is found to be at acceptable levels suggesting the potential for efficacy of well implemented SMC, a randomized control trial will be used to assess the effectiveness of different delivery systems to achieve the intended effect by improving coverage. The findings from years 1 and 2 implementation

studies will serve as the go (conduct high quality efficacy study) versus no-go (conduct further intervention effectiveness studies around operations and implementation strategies) criteria for years 3-5.

CRITIQUE 1

Significance:	2
Investigator(s):	2
Innovation:	3
Approach:	4
Environment:	2

Overall Impact:

Seasonal malaria chemoprevention (SMC) with monthly courses of sulfadoxine-pyrimethamine (SP) and amodiaquine targeting children under 5 years have been effective in significantly reducing malaria burden in sub Saharan Africa including that in Mali. Although SMC has made significant impact in controlling malaria, it has several logistic issues. In addition, it has been observed that there is an increase in the burden of malaria to older children and recent data from West Africa ICEMR suggests that there is a decreased reduction rate. Poor compliance to SMC may also contribute to the emergence of drug resistance. The proposed implementation (type 3) study aims to reduce malaria infection in children under 10. Studies are planned to test the hypothesis that SMC effectiveness in reducing malaria burden in Mali will depend on compliance to the dosage regimen and delivery mechanisms. These well designed studies led by a team of experienced investigators are aimed to perform a comprehensive analysis of SMC effectiveness during first two years. Although there is a high enthusiasm for this application, weaknesses are that Go/No-Go milestones are not clearly defined and there is some uncertainty about antibodies against AQ metabolite.

1. Significance:

Strengths

- The proposed research addresses an important problem relating to the effectiveness of SMC in Mali.
- There is an uncertainty about the SMC efficacy rates, which establishes the premise for the proposed study.
- The study plans to test the hypothesis that SMC success in Mali is influenced by delivery methods, patient compliance, and inclusion of children under 10. This is an important undertaking and the planned research would better define the utility of SMC in the management of malaria control.

Weaknesses

- None noted

2. Investigators:

Strengths

- Dr. Toure is trained as an epidemiologist who received his PhD from University of Copenhagen. He was the field epidemiologist of the Tulane ICEMR 2012-2017. Additionally, he has the experience in assisting Malian National Malaria Control Program (NMCP) for implementation of SMC.
- Dr. Schaffer is an experienced biostatistician who will be involved in data analysis and management. He is currently engaged in a similar role for a number of infectious diseases in West Africa.
- Dr. Barry is trained in malaria population genomics and she has a broad experience in various genomic techniques including, analysis of SNPs and whole genome analysis that would be relevant to this project.
- Dr. Cui is a very productive molecular parasitologist who recently was involved in the seminal study to develop ELISA-based quantification assay for artemisinin.
- The investigative team has complementary and synergistic expertise that is needed for a positive outcome for this project.

Weaknesses

- None noted

3. Innovation:

Strengths

- Innovation in this project comes from conducting a study to evaluate effectiveness of SMC in older children.
- Efforts to develop an ELISA based assay for detection of amodiaquine blood levels.

Weaknesses

- There is some uncertainty about the production of antibodies against the metabolite of amodiaquine

4. Approach:

Strengths

- The Aim 1 will be focused on executing rolling cohort studies with SMC targeting children up to 9 years of age and two health zones targeting children below 5 years. This study will establish the protective efficacy of SMC
- Definitions and measurements as well as outcomes have been clearly defined.
- Monoclonal antibodies against amodiaquine (AQ) metabolite N-desethyl-amodiaquine will be made to develop an ELISA using an approach similar to that recently developed for artemisinin.
- Evolution of any drug resistance SNPs will be determined. This is important because adherence to SMC is difficult and likely result in emergence of resistant mutants.
- Additionally, any changes in transmission dynamics will be evaluated.

- Sample size has been planned with attention to statistical considerations, which generates confidence to scientific rigor. The enrollment target of 1,000 each for children under 5 years and for under 10 years likely meets the required 2,000 individuals requirement.
- At the conclusion of the first 2 years of study, based on the outcomes of SMC efficacy two alternative paths have been proposed (1) efficacy studies or (2) effectiveness studies around operation and implementation policies.
- Years 3-5 proposed studies are well planned.

Weaknesses

- Although, Dr. Cui has been successful in developing an ELISA test for artemisinin, there is some uncertainty about getting specific antibodies against the AQ metabolite.
- Go-no go is rather a choice between two alternative routes of study in years 3-5 i.e., to conduct “efficacy studies” or conduct further “effectiveness studies around operations and implementation strategies”.
- Should there be widespread evolution of drug resistance in response to SMC, then Years 3-5 studies need to be discontinued. However, this is not a weakness of the application but a consequence of SMC efforts.
- Importantly, if it is possible to optimize SMC under budgetary constraints and if it would be feasible to bring the children 5-10 years under SMC coverage.

5. Environment:

Strengths

- Facilities and resources at University of Sciences, Techniques and Technology of Bamako (USTTB), Tulane University, and Walter Eliza Hall Institute are excellent for successful completion of the research proposed.

Weaknesses

- None

Study Timeline: UNACCEPTABLE
Comments: Go/no-go criteria are not well defined with quantifiable and measurable outcomes that will help in smooth transition to final 3 years of study.

CRITIQUE 2

Significance:	4
Investigator(s):	4
Innovation:	4
Approach:	6
Environment:	4

Overall Impact:

The project aims at assessing the effectiveness of the SMC approach for reducing mild and severe malaria cases in Mali. The descriptions in the application about what exactly will be done is a bit confusing and, in this review, it is assumed SMC for under-fives only is called no SMC. Clearly, finding the reasons for poor results of the existing program of SMC for under-five is an important public health issue but the evaluation needs to be design properly and conducted in places where the strategy is recommended to be implemented. It is not clear in the description of the suggested areas for evaluation that this is the case i.e. the SMC is being implemented in areas where transmission is longer than 3-4 months. Furthermore, effectiveness of the SMC is proposed to be evaluated in individually randomized trial while the interest is on whether community delivery of the intervention is providing the benefit to the community overall as the large implementation trials have demonstrated and hence the WHO recommendation. The recommendation is for the group to consider design a cluster randomized trial of current SMC compared with SMC extending to age 10 yrs. Also use the first 2 years to assess the coverage issues and pilot the suggestions of improvements in delivery mechanisms of the SMC.

1. Significance:

Strengths

- The application addresses an important issue related to malaria control in the Sahel region and finding solutions for optimization of SMC strategy is critically important
- Should the work show that extension of the SMC is cost effective it will change the implementation of this approach in Mali and possibly the rest of the Sahel region
- The team have already links and had done some work with the Malaria Program and hence the findings in this will easily be taken for scale up in the country

Weaknesses

- SMC impact can vary due to many factors and background information about what are the existing difficulties with the current implementation is generally lacking and the proposed work is not focused on few specific issues to be tackled.
- The data to justify change of approach need to be based on scientifically credible evidence based on rigorous design and implementation at community level. The application has not described well the evaluation design and include the individual randomized approach which may not be appropriate for this assessment.

2. Investigators:

Strengths

- There is strong and capable local team supported by equally capable international collaborators with various expertise from epidemiology to laboratory sciences.

Weaknesses

- None

3. Innovation:

Strengths

- The application to extend SMC intervention up to 10 year old children is innovative and some studies are ongoing and this will add to the body evidence to advocate this approach
- The use of simple technology for assessment of drug levels with community samples as well as use of the more technologically advanced molecular epidemiology techniques will advance our understanding of both the implementation and impact or adverse outcomes in terms of resistance of the SMC program

Weaknesses

- Although new technologies will be used for assessing drug levels and resistance it is not clear in the application how this information will be used to translate into better implementation and impact of the SMC approach

4. Approach:

Strengths

- The application proposes to assess what are the issues with the implementation of the SMC first then decide on the appropriate trial to conduct to show impact of SMC
- The use of existing established areas where the team has been working make it easier to set up

Weaknesses

- The design need to select areas where the SMC intervention has the highest impact. It is not clear whether irrigated areas where transmission may be longer than 4 months will be appropriate.
- The design currently suggests comparison of 2 types of zones and those that have SMC and those that have not. It is not clear how the decision to provide SMC or not was made or will be decided. Furthermore, this will be a comparison of one area with another without SMC. It may well be the reasons for selection determine the coverage and performance of the SMC.
- For the effectiveness evaluation an individually randomized trial is proposed while the appropriate approach is to conduct a community cluster randomized trial. Furthermore, the comparison should be the made between benefits of SMC under five only as compared to SMC for under 10 years. It is highly unlikely that there will be a discussion about using SMC for those 5-9 only.
- Details of how the end points data will be collected, quality assured is missing and this will need to be added in the application.

5. Environment:

Strengths

- The team has been working with the Malaria control program and this will facilitate discussions about the suggested improvements in the implementation of the strategy

Weaknesses

- The involvement of implementation partners is not well described as well as the exact details of the implementation processes in the different areas

Study Timeline: ACCEPTABLE

CRITIQUE 3

Significance:	3
Investigator(s):	2
Innovation:	4
Approach:	4
Environment:	2

Overall Impact:

The proposed sequence of studies have strong potential to provide substantive research on whether the disappointing effectiveness of the SMC approach to malaria control is related to implementation or lack of effectiveness in the setting. The proposed interventions seem pragmatic and implementable, and thus have the potential to exert considerable impact on malaria in Mali. The team is experienced, with evidence of strong collaboration. There is an element of innovation, based on the inclusion of the resistance and transmission dynamics work, developed by the Asia Pacific ICEMR. My enthusiasm is tempered by the lack of detail in the study designs, the confusion about what the primary questions are and a lack of clarity about how data generated will answer the hypothesis: generally there appears to be a lack of coordination between the proposed studies and the questions they are designed to address.

1. Significance:

Strengths

- The sequence of studies is well designed to achieve a good understanding of the underlying issues that affect effectiveness of SMC in these regions of Mali, and evaluate one of two different testable strategies for improving effectiveness – either through delivery mechanisms or increased age group targeting.
- The team, from this new ICEMR, has been impressive in generating preliminary data about the disappointing effectiveness of SMC in Mali, leading to the hypotheses presented.
- Well positioned to provide critical information about the SMC approach to reducing malaria.

Weaknesses

- A concern is that the districts may not meet the conditions suitable for SMC intervention, as they are described as having “virtually year-round malaria transmission”, so the reason for lower effectiveness from the seasonal approach may be the lack of seasonality. This possibility was not discussed.

2. Investigators:

Strengths

- Toure proposed as leader of Project 2: Recent PhD (graduated?) in Epidemiology. ~5 years experience in field epidemiology, in Mali, with current role as Data management CORE leader in ICEMR.
- Senior personnel Kayentao, S Diakite: Both have solid experience in field implementation.
- In publications, strong evidence of collaboration between members of the group

Weaknesses

- This is a much larger field study than previous leadership roles for Toure, although Dr Doumba will provide oversight. [minor]
- The lack of clarity between the design and the planned hypotheses and their evaluations leads to concern about the integration and communication between the clinical investigators and the team's statisticians. In Aim 3 the two different types of interventions and designs are muddled in the application, and references Tables are missing. [Major]
- The team effort is far flung: Mali, Tulane, Australia, Denmark, Penn State. [Minor]

3. Innovation:

Strengths

- Inclusion of the SNP work for studying parasite population structure has the potential to be very informative about malaria epidemiology
- Aim 3 cluster randomized trials have the potential to be definitive trials with regard to efficacy of SMC.

Weaknesses

- None noted

4. Approach:

Strengths

- The sequence of trials in the Years 1-2 and the alternatives for year 3-5, depending on outcomes from Years1-2 are sensible and well described. The Go-No go criteria for selecting the intervention to evaluate in the subsequent study are appropriate, albeit not precisely defined.
- There are sufficient communities to conduct an adequately community randomized trial for the Alternative Aim 3 design. The choice of household randomization for the Aim 3 design is good from a design point of view, although it is not clear how SMC would be implemented differently on a household-by-household basis.

Weaknesses

- The evaluations and hypotheses planned for the cohort and cross-sectional study are unclear and confusing. The primary exposure variable (SMC exposed and non-SMC-exposed children) is not defined. All communities are receiving SMC as the standard of care, and if it is planned to use self-report of SMC-exposed, the sample size does not mention how the proportion of exposed children factors into study power, or how time

varying measures of SMC exposure will be used. Similarly, if there is any plan to evaluate whether child age is an important factor in SMC implementation it is not clear how that will be evaluated in the planned initial studies.[Major]

- There is no description of how children or households would be selected for the cohort or cross sectional study. [Minor]
- The use of the two different age group exposures to SMC in the 4 communities is not explained. The approach appears to describe only assessment of children < 5 yo in both the cross sectional and cohort study, so there would be no measurement of the SMC effect in the older age-group [Major]
- There are discrepancies between the approach and stated hypotheses and the sample size/analysis sections in what the primary assessments goals are. E.g. “The sample size assessment is based on the expected difference in cumulative incidence of uncomplicated malaria between SMC-exposed and SMC-unexposed children under 10 years of age.” However the cohort is only planning to enroll children < 5 years old.[Major]
- The hypothesis, as described, for Aim 3 is an equivalence trial. The sample size for this hypothesis are not appropriate (appears to be the for the alternative design)[Major]
- In the study of transmission dynamics, it is not described how a control population is defined from the cross-sectional survey, where all households are in districts receiving SMC. [Major]

5. Environment:

Strengths

- Clearly identifies personnel qualified to conduct data collection, laboratory and administrative function
- Demonstrated ability and experience in conducting trials in this setting
- Letters of support from MOH and NIAID, the implementing partners for SMC

Weaknesses

- None noted

Study Timeline: ACCEPTABLE

CRITIQUE 4

Significance:	2
Investigator(s):	2
Innovation:	3
Approach:	7
Environment:	2

Overall Impact:

Malaria prevalence and disease incidence remain high in Mali and in many other countries in Sub-Saharan Africa despite the use of an array of malaria interventions. This study has the potential for high

overall impact by conducting research to determine the long term impact of SMC on acquired immunity, parasite transmission dynamics, drug resistance, and clinical outcomes in children less than 5 years of age and in children 5-9 years of age, thus expanding the age for SMC administration. Expanding the age group for SMC is of particular interest given the shift in malaria burden from children under five to older children. The significance of this work is high, and the investigators in Mali have established global collaborations to bring in technical expertise, which contributes to the likelihood of success. However, the approach was unclear and needs to be further clarified in terms of establishing the two age groups of under and over fives in the intervention and control groups. Specifically, it was unclear how the investigators will be establishing a no intervention group in children under five given SMC is national policy. In addition, there are not clear Go/No-Go criteria. If the milestones from phase 1 are not met the investigators propose an alternate aim 3, which is not the same as a No-Go.

1. Significance:

Strengths

- Malaria interventions are having less impact on malaria burden and disease than expected and this project has the ability to evaluate one of these interventions (SMC) and its long term effects on acquisition of immunity, parasite populations, and clinical disease, as well as to assess how well the intervention is being implemented (e.g. compliance). The project looks at the impact of SMC on multiple outcomes beyond just looking at clinical disease.
- Investigating the impact of an intervention targeting older children (5-9 years) is highly significant give the shift in burden of malaria from children under five to those over five.
- The cost effectiveness information will help the National Malaria Control Program and its partners in determining which interventions to utilize in the face of limited funding.

Weaknesses

- None.

2. Investigators:

Strengths

- This project is led by a strong team of researchers at USTTB in Mali. The PD is in the early stages of an independent career, but has appropriate training and experience.
- The team in Mali has established a strong partnership with researchers at Tulane University for over the past 10 years. Investigators from Tulane University ran the previous West African ICEMR. In addition, this project has brought in global technical expertise in multiple areas, including determining amodiaquine levels in the blood, parasite population genomics, and drug resistance markers, some of which are from other ICEMRs.

Weaknesses

- None.

3. Innovation:

Strengths

- This will be one of the first studies to assess SMC in children over 5 years of age.

- This project utilizes a new method for determining amodiaquine levels in the blood as a measure of compliance in collaboration with the Southeast Asia ICEMR.
- The project also utilizes a new system for assessing parasite population genomics as developed by the Asia Pacific ICEMR.

Weaknesses

- None.

4. Approach:

Strengths

- The project uses a variety of methods including a cohort study to assess long-term impacts, a cross sectional survey to assess coverage, and a randomized control trial to assess the impact in children over five years of age.
- This project will provide an interesting look at impacts on parasite genomics and the investigators have established strong collaborations to conduct these analyses.

Weaknesses

- The investigators propose to study the effectiveness of SMC by age group (<5, 5-9) for SMC vs. no SMC. Given SMC is national policy it is unclear how they will get a group of children under 5 without SMC. Will this be the children under five who never got SMC for whatever reason? If this is the case there will be other confounding factors that need to be taken into account. Or will this be comparing communities where only children under five get SMC vs. communities where children under five and 5-9 get SMC, which means the under-fives are actually getting SMC? The description of the approach seems conflicting in that in one sentence it says all under fives will get SMC and then it refers to the outcomes in under-fives without SMC. In addition, more details are needed on the randomization and administration of the SMC to children 5-9 years of age in years 3-5.
- The application is missing tables outlining the sampling, etc. that are referred to in the text, which may have assisted in understanding the different age groups and intervention vs. no intervention arms.
- The investigators have a plan B for years 3-5 if the results of years 1-2 suggest that SMC is not being well implemented. Instead of moving forward with the efficacy study, they will conduct implementation research to improve upon the administration of SMC. So while it is commendable that they have planned ahead this does not qualify as a true Go/No-Go criteria. The Go/No-Go criteria is designed so that if phase 1 milestones are not met the project does not move forward. Here the investigators are proposing to still move forward, but in another direction.
- For alternative aim 3, the investigators need to provide the justification for repeating the fixed point vs. door-to-door delivery system study. The only new factor seems to be the expanded age group.
- Measuring compliance relies on the ability to develop the monoclonal antibodies and icELISA, which are not already available at the outset of the project. It is noted that the investigators include a team with experience in preparing these so the risk is limited making this only a minor weakness.

5. Environment:

Strengths

- The West African ICEMR in Mali has developed a linkage with the Asia Pacific ICEMR to receive support for the parasite population studies and the Southeast Asia ICEMR for determining amodiaquine levels in the blood.
- USTTB is a longstanding research center in Mali.
- Collaborators from around the world will bring expertise for some of the studies and analyses including parasite population genomics, ELISA for amodiaquine in the blood of children receiving SMC, and analysis of drug resistance markers.

Weaknesses

- None.

Study Timeline: UNACCEPTABLE
Comments: Instead of a No-Go decision if the milestones are not met in the first two years, the investigators propose an alternate line of investigation for years 3-5.

THE FOLLOWING RESUME SECTIONS WERE PREPARED BY THE SCIENTIFIC REVIEW OFFICER TO SUMMARIZE THE OUTCOME OF DISCUSSIONS OF THE REVIEW COMMITTEE ON THE FOLLOWING ISSUES:

PROTECTION OF HUMAN SUBJECTS (Resume): ACCEPTABLE (Code 30)

- Project 2 Comments:
  - While the Investigators describe very well Ethical considerations for this study and indicate that they plan to obtain written Informed Consent, in the section describing the process on P181, they indicate that it is anticipated that many respondents will be non literate or unable to speak or read French and that in such cases, "……it will be necessary to read the consent form slowly and obtain verbal consent."
  - It is unclear how reading the form slowly in a language they do not understand will make the respondents understand the consent form and why investigators cannot explain the contents of the form in a language they can understand and allow them to thumbprint to indicate consent if they cannot sign for some reason.

INCLUSION OF WOMEN PLAN (Resume): ACCEPTABLE (Code 1A)

INCLUSION OF MINORITIES PLAN (Resume): ACCEPTABLE (Code 1A)

INCLUSION OF CHILDREN PLAN (Resume):

Project 1 ACCEPTABLE (Code 3A)

Project 2 ACCEPTABLE (Code 2A)

VERTEBRATE ANIMALS (Resume): NOT APPLICABLE (CODE 10)

BIOHAZARD COMMENT: ACCEPTABLE
Biohazards, if present, are named and appropriate responses and handling procedures given.

FOREIGN INSTITUTION: JUSTIFIED

- Comments:
  - Mali is proposed for the field studies. This is justified as malaria is endemic there. Expertise from other institutions is important for the successful implementation of the proposed study. The collaboration is justified.
  - This ICEMR, including the supplementary pair of projects is a superb, strategic long-term investment in a Malian institution that is far better placed to tackle malaria over the long term than any of the partners institutions in non-endemic countries.

SELECT AGENTS: NOT APPLICABLE

DATA SHARING PLANS: ACCEPTABLE

MODEL ORGANISM SHARING PLANS: NOT APPLICABLE

GENOMIC DATA SHARING PLAN: NOT APPLICABLE

AUTHENTICATION OF KEY BIOLOGICAL AND/OR CHEMICAL RESOURCES: ACCEPTABLE

BUDGETARY OVERLAP: ACCEPTABLE

COMMITTEE BUDGET RECOMMENDATIONS: ACCEPTABLE

- Project 1 Comments: Fine, although I'd encourage greater contributions to core and administrative functions as direct costs to maximize sustainability of the Malian prime recipient.

Footnotes for 3 U19 AI129387-03S1; PI Name: DOUMBIA, SEYDOU

NIH has modified its policy regarding the receipt of resubmissions (amended applications). See Guide Notice NOT-OD-14-074 at http://grants.nih.gov/grants/guide/notice-files/NOT-OD-14-074.html. The impact/priority score is calculated after discussion of an application by averaging the overall scores (1-9) given by all voting reviewers on the committee and multiplying by 10. The criterion scores are submitted prior to the meeting by the individual reviewers assigned to an application, and are not discussed specifically at the review meeting or calculated into the overall impact score. Some applications also receive a percentile ranking. For details on the review process, see http://grants.nih.gov/grants/peer_review_process.htm#scoring.

MEETING ROSTER

National Institute of Allergy and Infectious Diseases Special Emphasis Panel
NATIONAL INSTITUTE OF ALLERGY AND INFECTIOUS DISEASES
Limited Competition: Revision Applications for International Centers of Excellence for Malaria Research
(U19 Clinical Trial Optional)
ZAI1 AMC-M (J1)
09/26/2018 - 09/28/2018

Notice of NIH Policy to All Applicants: Meeting rosters are provided for information purposes only. Applicant investigators and institutional officials must not communicate directly with study section members about an application before or after the review. Failure to observe this policy will create a serious breach of integrity in the peer review process, and may lead to actions outlined in NOT-OD-14-073 at https://grants.nih.gov/grants/guide/notice-files/NOT-OD-14-073.html and NOT-OD-15-106 at https://grants.nih.gov/grants/guide/notice-files/NOT-OD-15-106.html, including removal of the application from immediate review.

[redacted]

[redacted]
